# High moisture confluence in Japan Sea polar air mass convergence zone captured by hourly radiosonde launches from a ship

**DOI:** 10.1038/s41598-022-23371-x

**Published:** 2022-12-23

**Authors:** Yoshihiro Tachibana, Meiji Honda, Hatsumi Nishikawa, Hiroaki Kawase, Haruna Yamanaka, Daichi Hata, Yuji Kashino

**Affiliations:** 1 Faculty of Bioresources, Mie University, Tsu, Japan; 2 Faculty of Science, Niigata University, Niigata, Japan; 3 Atmosphere and Ocean Research Institute, The University of Tokyo, Kashiwa, Japan; 4 Meteorological Research Institute, Japan Meteorological Agency, Tsukuba, Japan; 5 Japan Fisheries Research and Education Agency, National Fisheries University, Shimonoseki, Japan

**Keywords:** Ocean sciences, Physical oceanography, Natural hazards, Cryospheric science, Ocean sciences, Physical oceanography, Climate sciences, Atmospheric science, Atmospheric dynamics

## Abstract

Some of the heaviest snowfalls in urban areas in the world occur in Japan, particularly in regions that face the Japan Sea. Many heavy snowfalls are produced by a Japan Sea polar air mass convergence zone (JPCZ), which is an atmospheric river-like cloud zone that forms when Siberian cold air flows over the warm Japan Sea. Quantifying how the air–sea interaction strengthens the JPCZ is key to snowfall prediction. However, until our observations with hourly meteorological balloon launches from a training vessel in 2022, no simultaneous air–sea observations targeting the JPCZ had been conducted. Our observations showed that wind direction shifted drastically by about 90 degrees from the surface to an altitude of about 3.5 km within a narrow horizontal range of about 15 km, indicating airflow convergence from the surroundings. Maximum temperature difference between surface air (3 °C) and water was 11 °C near the JPCZ centre with 17 m s^−1^ wind speed. Large amounts of heat, 718 W m^−2^, was thus gained from the warm sea. Water vapour was also concentrated by the horizontal convergence, which caused heavy snow, equivalent to 100 cm of snowfall in 7 h. The surrounding sea greatly affects moisture formation within the JPCZ.

## Introduction

**Figure 1:**
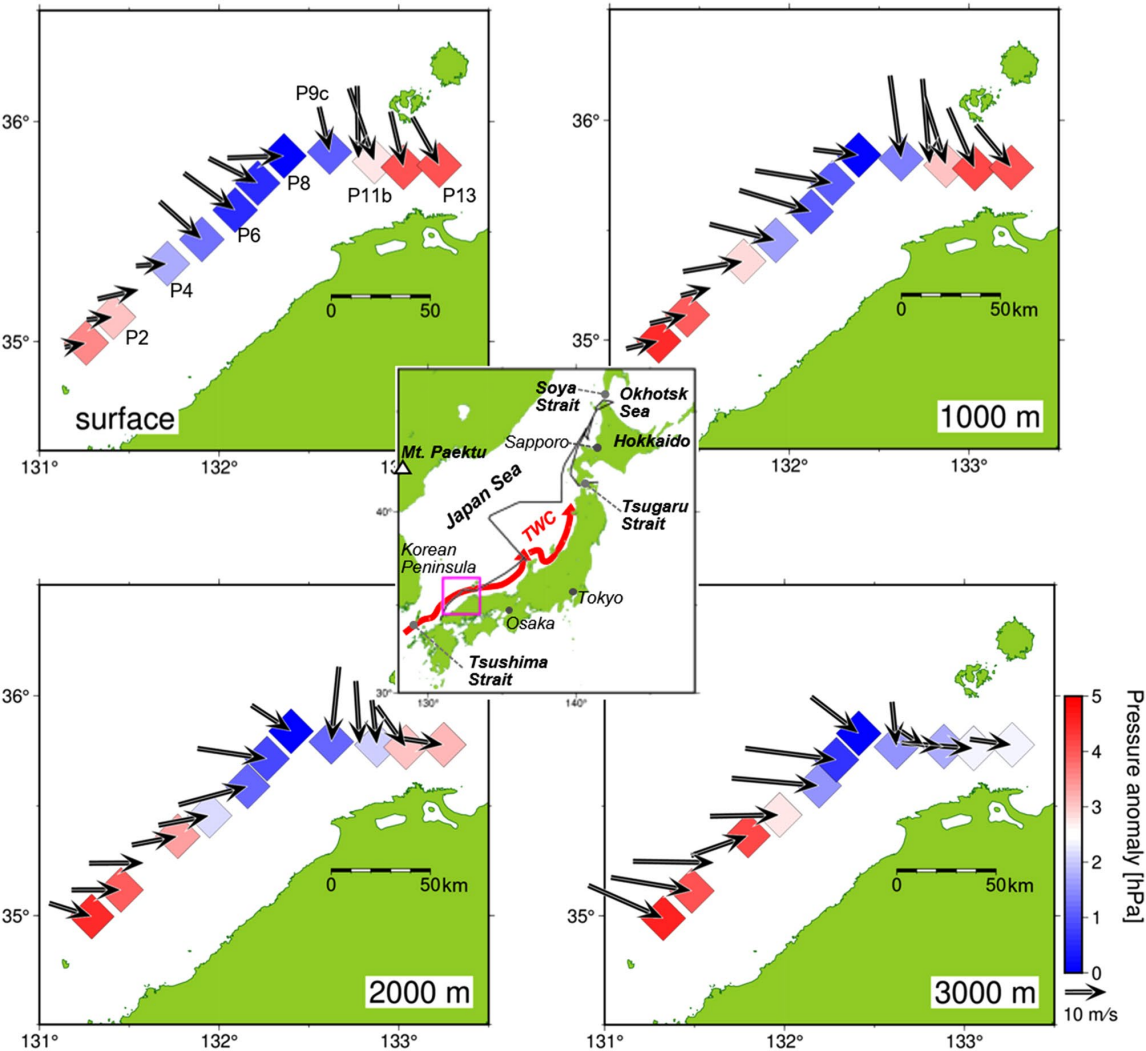
Drastic changes in wind direction at the centre of the JPCZ. (Centre map) Observation area shown in panels (enclosed in red box) and the observation transect (black line). An arrow marked TWC indicates the Tsushima Warm Current. (Observation area) Horizontal map of wind direction and speed at surface and altitudes of 1000, 2000, and 3000 m along 1-h-interval radiosonde launches. Even-numbered observation stations (P2, P4, etc.) are shown in the upper left map (surface). Colours of individual squares representing observation stations indicate the air pressure anomalies from the minimum pressure at each altitude. The minimums were located at station P8 for all altitudes. Colour bars bluish and reddish colours indicated lower and higher anomalies, respectively.

Winter weather in some plains regions of Japan facing the Japan Sea is distinguished by receiving deep snow accumulation^1^. Some of these heavy snowfall areas are urban areas, such as Sapporo (population 2 million, Fig. 1), where the maximum snow depth in a winter is as much as 100 cm^2,3^. In these regions, as much as 100 cm of snow can occasionally accumulate in a single day^4^. In contrast, in the regions facing the Pacific Ocean, such as Tokyo, snowfall events rarely occur. Although the deep snow accumulations have led to the development of unique regional culture and provides a stable water resource, disruptions from heavy snowfall occur every winter. For example, the super express Shinkansen trains connecting Tokyo and Osaka and superhighways are often closed due to heavy snowfall. Thus, understanding and predicting heavy snowfall events is important. From meteorological satellite images, the cause of heavy snowfall has been roughly identified as being the occurrence of a strong cloud formation extending in a band, which is referred to as the Japan Sea polar air mass convergence zone (JPCZ)^5^. The JPCZ extends from the base of the Korean Peninsula to the Japanese archipelago and is about 1000 km long. The location of JPCZ initiation is always the same, downstream of a high mountain, Mt. Paektu, at the northern root of Korean Peninsula^6^. The areas where the JPCZ makes landfall experience much heavier snowfall than surrounding areas^4^. The shape of the JPCZ resembles a miniature atmospheric river^7,8^ complete with narrow bands of enhanced water vapour transport. Previous numerical simulations showed that JPCZ initiation is caused by the confluence of two sets of the airflows that go northward and southward around Mt. Paektu^9^. However, no direct observations of this confluence or the strength of this confluence have been made. In the case that this confluence is occurring, vertical and horizontal characterization of the JPCZ is needed to confirm horizontal convergence that can produce an updraft sufficient to produce the strong convective clouds found in the JPCZ. Moreover, no observational studies to quantify water vapour condensation heating in the JPCZ to evaluate the buoyancy, updraft, and strength of the JPCZ have not been conducted. These important unsolved problems can be tackled by direct shipboard observation.

Because the airflow that goes around Mt. Paektu originates in Siberia with prevailing winter monsoon winds, the air over the Japan Sea is expected to be cold. On the other hand, the Japan Sea is warm because a part of the warm current enters through the Tsushima Strait into the Japan Sea^10^, which originates from the Kuroshio^11^. This current is referred to as the Tsushima Warm Current. The behaviour of the Tsushima Warm Current is also expected to play an important role in the development of the JPCZ because the warm sea supplies the overlying cold airmass with large amounts of water vapour and heat^9,12–16^. The heated surface airmass has the potential to weaken atmospheric vertical static stability because cold air with high density overlies the warm surface air with low density. Thus, deepening our knowledge of the spatial and temporal variations of the Tsushima Warm Current is also important. Furthermore, the overlying atmosphere influences sea surface temperature (SST) variation^17^. Thus, SST variation is influenced by interaction of the air–sea behaviours of both the Tsushima Warm Current and the overlying atmosphere^15^. Despite simultaneous observation of the atmosphere and ocean provide a close-up examination of their interactions and deepen our understanding of the mechanism of the JPCZ, no atmosphere–ocean observations of the JPCZ have been made.

The JPCZ does not always form when Siberian cold monsoon winds flow to the Japan Sea. A typical cloud pattern in the period of the winter monsoon winds is a packet of ‘normal’ band-shaped clouds without the appearance of JPCZ. These clouds take the form of a cloud street, as seen in the Great Lakes region of North America^18^, but it is not as distinctive as the JPCZ. Another typical cloud pattern is a cyclonic vortex referred to as a polar low, which occasionally appears over the northern part of the Japan Sea^19^. What conditions determine the formation of the JPCZ rather than other formations? To answer this question, we conducted direct observations of both the JPCZ and other cloud formations.

We conducted air-sea simultaneous observations targeting the JPCZ from the training vessel, Koyo-maru, from January 19 to 20, 2022. The ship successfully traversed the JPCZ, and meteorological balloons were launched at 1 h intervals. At the same time, vertical profiles of salinity and temperature in the ocean were measured. The vessel moved from the southern edge of the Japan Sea to the northern edge until the end of January (Fig. 1) and measured a variety of air-sea interactions covering a wide area of the Japan Sea, where ‘normal’ band-shaped clouds and polar lows were observed. This paper quantitatively describes, for the first time, airflow confluence, horizontal convergence of wind and water vapour, condensation heat in clouds, and precipitable water of the JPCZ along with the oceanic role. First, we show the structure of the JPCZ based on underlying oceanic conditions. Next, we provide an overview of the ‘normal’ band-shaped clouds and polar low observations compared to those of the JPCZ. Based on the comparison, we propose a possible oceanic role for the frequent occurrence of JPCZ and polar lows over the Japan Sea. Third, a brief comparison of the observed values with those of a numerical simulation is provided. Finally, we estimate the magnitude of the horizontal moisture convergence and upward heat and moisture flux from the underlying ocean. Based on these estimations, we estimate the snowfall capacity of the JPCZ. Publication of atmospheric and oceanic observation data in this paper will contribute to the validation of numerical experimental studies and to improving air-sea reanalysis projects and simulations of the JPCZ and the Japan Sea atmospheric conditions.

## Results

### Sharp horizontal convergence in the centre of JPCZ

**Figure 2:**
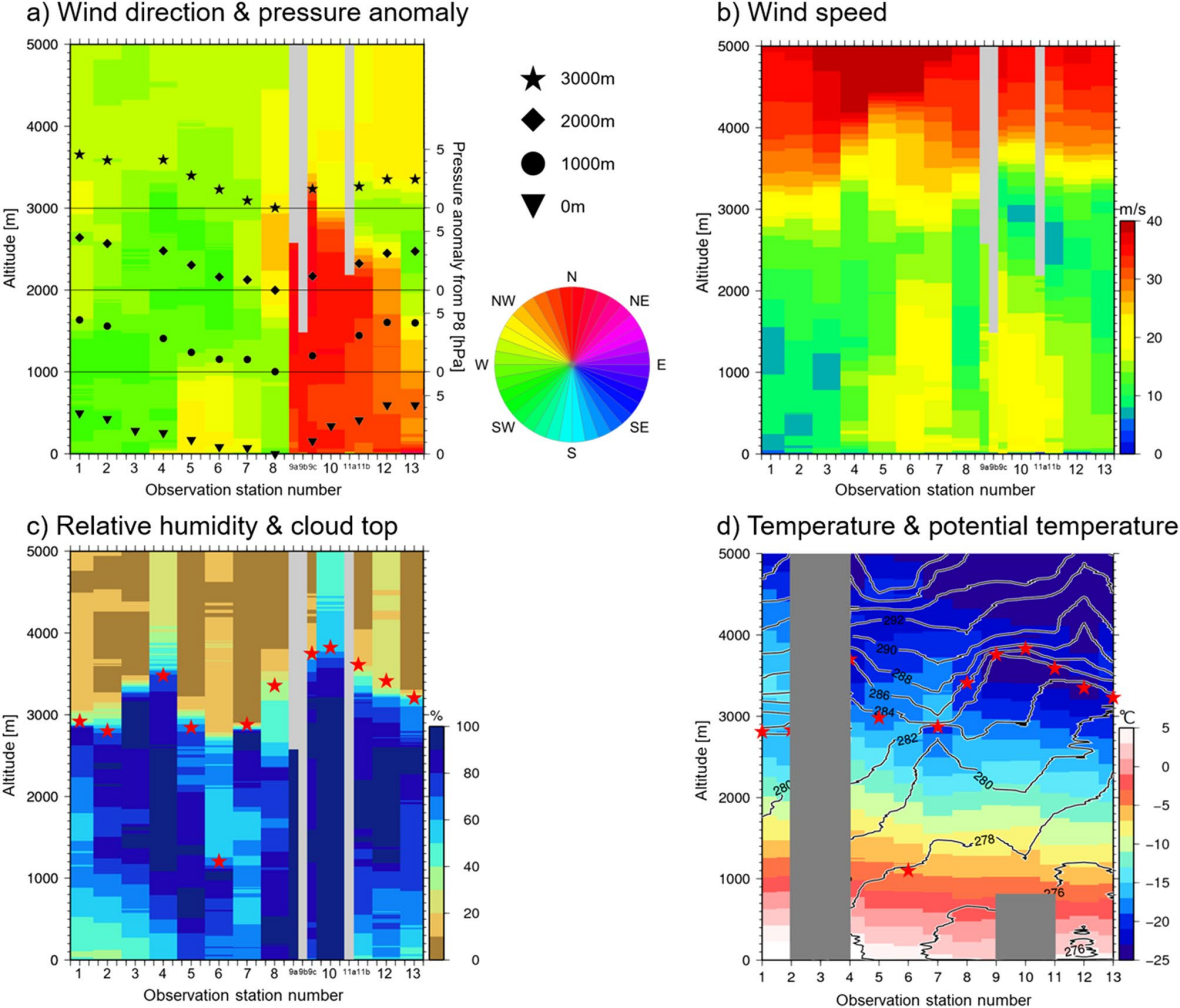
Horizontal airflow convergence reached altitudes of 3.5 km in association with wind direction changes. Atmospheric horizontal-vertical cross sections along the observation stations. (**a**) Colour shows wind direction. Reddish and greenish indicate north and west winds, respectively, as shown in the pie chart. Triangle, circle, square and star symbols are pressure anomalies at 0, 1000, 2000, and 3000 m, respectively. The anomaly is deviation from observation station P8, at which minimum pressure was recorded. The scale of the pressure anomaly is indicated on the right y-axis. Horizontal thin lines are zero lines for the pressure anomalies at each altitude. (**b**) The same as in (**a**) but for wind speed. (**c**) The same as in (**a**) but for relative humidity. Star symbols show cloud top heights. The cloud top altitude was determined from the agreement between the temperature significant level and altitude of the rapid decrease in relative humidity to below 85%^20,21^. (**d**) Colours indicate temperatures, and contours show potential temperature. Star symbols show the heights of temperature inversion layers. Others are same as in (**a**). Gray indicates missing data.

The horizontal wind direction and speed at surface and at altitudes of 1000, 2000, and 3000 m determined in 1-h intervals by radiosonde launches near the western coast of Japan are shown in Fig. 1. The horizontal-vertical cross sections of wind direction and speed are shown in Fig. 2a,b. Wind direction differed by about 90 degrees between stations P8 and P9a where the two air flows appeared to be confluent over a horizontal distance of 15 km and an altitude of about 3.5 km. Minimum air pressure anomaly readings were recorded at P8 at all altitudes sampled. From this drastic change in wind direction and the accompanying pressure minimum, we determined that the centre of the JPCZ was located between P8 and P9a. By contrast, there was no such drastic change in wind speed (Fig. 2b), although the wind speed minimum was located at P8, indicating a decrease in horizontal wind speed nearer to the centre. This decrease signifies the presence of an updraft associated with horizontal airflow convergence as much of the confluent air masses must charge the updraft. The radiosonde observations thus captured horizontal airflow convergence from surrounding areas. The horizontal distance between two adjacent points where the wind direction changed drastically corresponds to the width of a convergence zone, which was about 15 km. Because the convergence was up to 3.5 km in height, an updraft to this altitude was expected to be observed. The deep convergence also implies that updraft speed is greater at higher altitudes. Documentation of the convergence to an altitude of 3.5 km is quite unique. The sharp decrease in relative humidity with altitude appears at about 3.5 km height (Fig. 2c), indicating the cloud top height near the centre of the JPCZ^20,21^. Cloud height observed in other ‘normal’ clouds is usually 1.9 km, indicating the JPCZ cloud top to reach nearly twice as high as usual clouds. The temperature inversion layer capped the cloud top near the centre of the JPCZ (Fig. 2d). Vertical stratification, which is expressed by the vertical gradient of potential temperature, was weak near the centre from the surface to the cloud top (Fig. 2d). These temperature fields are consistent with the presence of the expected updraft. It should also be noted that the temperature field did not show such a drastic change as was present for wind direction. Thus, no horizontal temperature front associated with the drastic wind change was observed.

**Figure 3:**
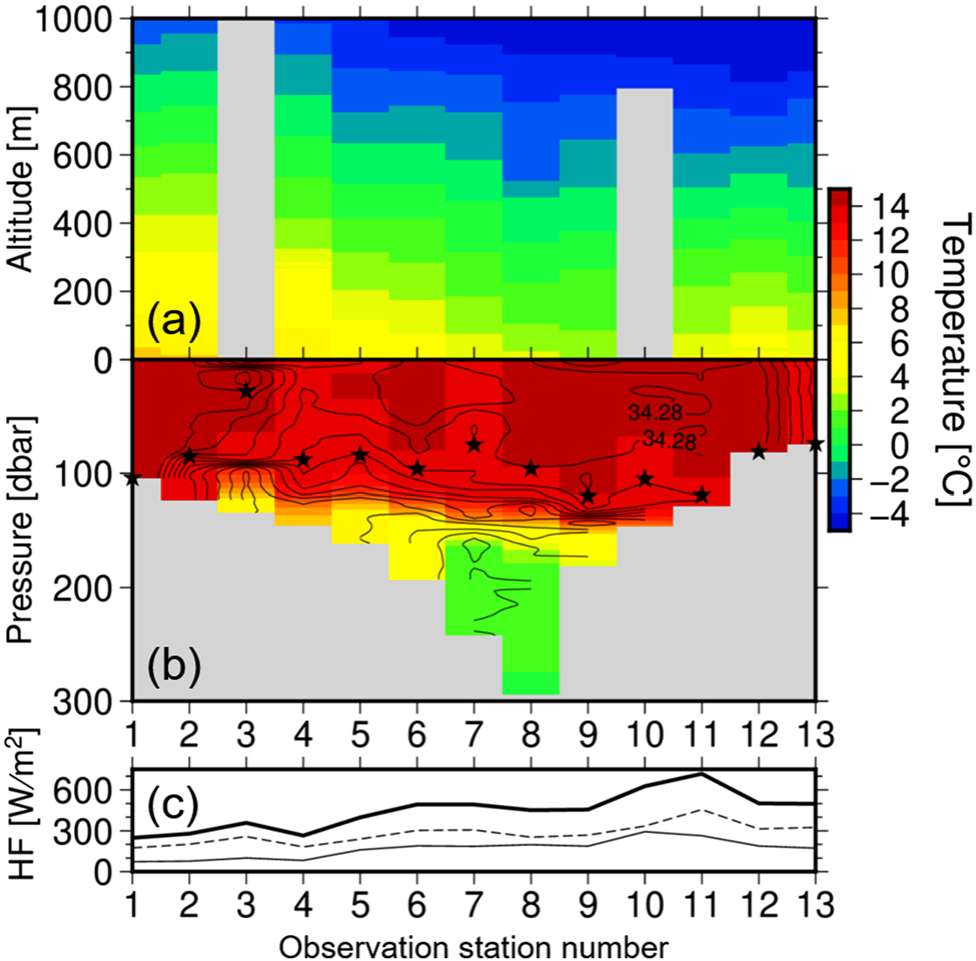
Ocean near-surface temperatures were much higher than overlying atmosphere under the JPCZ. (**a**) Vertical cross sections of atmospheric temperature and (**b**) underlying oceanic temperature along the observation stations. To make the atmosphere–ocean interaction easier to see, the colour intensity was kept the same for both atmospheric and oceanic temperatures as indicated on the right side of the figure. Contours in the ocean denote salinity with interval of 0.02. Stars indicate ocean mixed layer depth defined as the depth at which density increases by 0.03 kg m^−3^ from its 10-dbar depth value^23^. (**c**) Sum of latent and sensible heat fluxes from the ocean to the atmosphere. Positive value means upward flux. The thin, broken, and bold lines indicate sensible, latent, and total heat flux, respectively.

Figure 3 shows the horizontal-vertical cross section for atmospheric temperature and underlying oceanic potential temperature near the surface. While air temperature tended to gradually decrease with increasing observation station number, oceanic temperature was overall uniform due to the mixed layer of the ocean. That is, no synchronising signature between the air and sea is observed. This suggests that atmospheric temperatures near the sea surface were not strongly influenced by local changes of underlying sea temperatures, which were much higher than those of the overlying atmosphere. SSTs under the JPCZ area were on average 14.0 °C, while overlying surface air temperature was on average 5.5 °C. This large temperature difference along with strong wind speeds supplies the air mass with a large amount of heat from the ocean. Maximum heat flux was observed at P11, where air temperature and SST were 3.0 and 14.0 °C, respectively, with a temperature difference of 11.0 °C. Because of this large temperature difference between the atmosphere and the ocean, and surface wind speed of 17 m s^−1^ at P11, the estimated oceanic heat flux was 718 W m^−2^, where both latent and sensible heat fluxes were calculated by a bulk method^22^. The heat flux at P11 comprised 455 W m^−2^ of latent heat and 263 W m^−2^ of sensible heat. The average heat flux was 445 W m^−2^ in the JPCZ region. Interestingly, the heat flux in the centre of the JPCZ at P8 was not as large as at P11 because wind speed in the centre was weaker than at P11 (Fig. 2b). Smaller temperature difference between air and sea surface at P8 than at P11 was also responsible for the small heat flux at P8. It should therefore be noted that water vapour in the centre of the JPCZ was supplied not only from the underlying sea but also from surrounding areas with strong convergent wind. The mixed layer depth^23^ of the ocean was overall the same along the transect at about 88.8 m. Salinity in the mixed layer was also uniform overall, except at P13. The deep mixed layer, which has a large heat content, is unlikely to have a change in SST, even if a large amount of heat escapes to the atmosphere. Heat release of about 400 W m^−2^ has the potential of lowering the water temperature at a depth of 100 m by only about 0.1 °C per day at most. As a result, the underlying sea can continue to supply heat and moisture to the atmosphere, even if the JPCZ repeatedly forms there.

### Overall atmospheric and oceanic structure over the Japan Sea and comparison of JPCZ with the others

**Figure 4:**
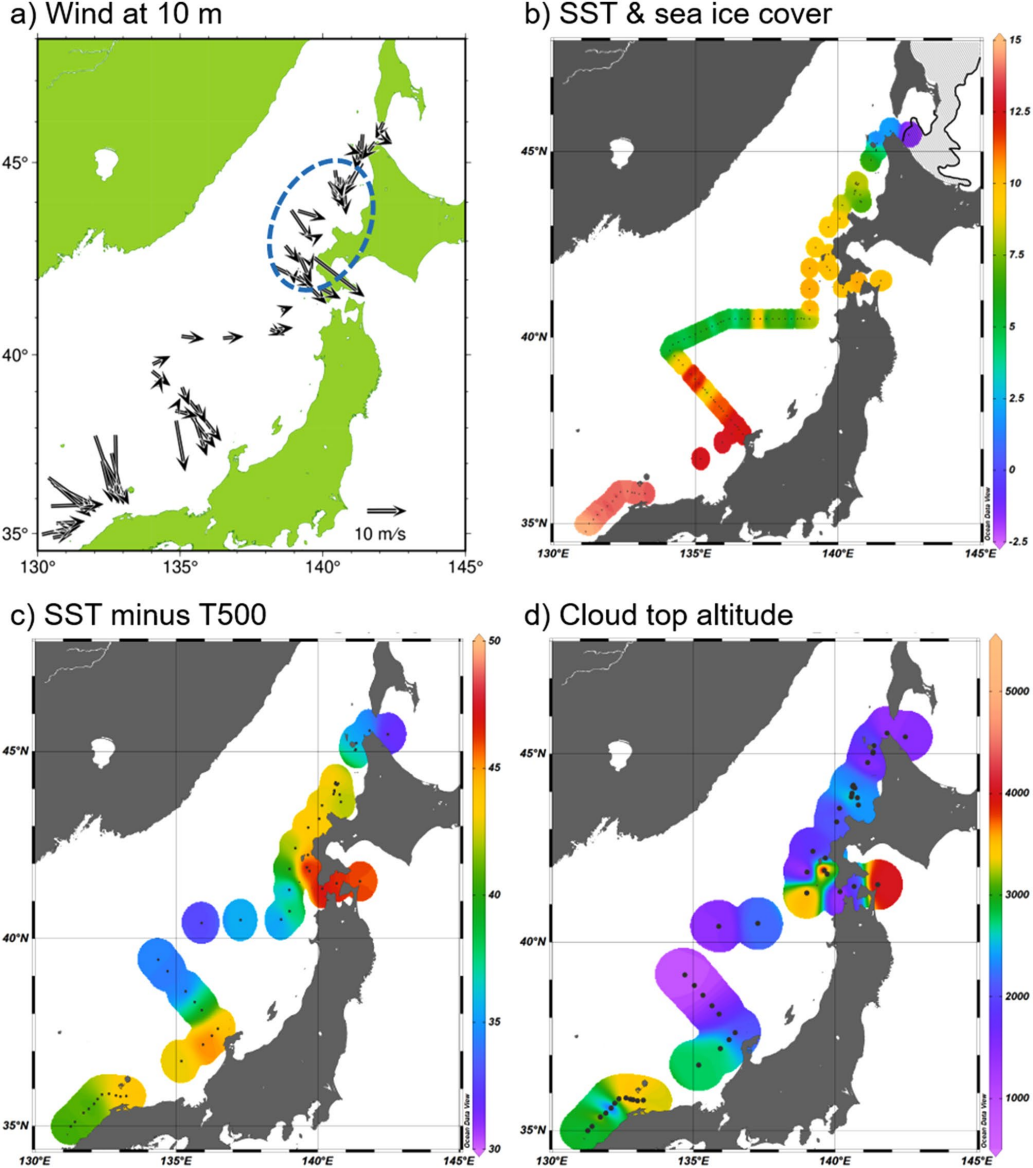
Observed air sea conditions during entire observation period. (**a**) Observed wind vector at altitude of 10 m. In addition to the JPCZ area, the vortex-like wind distribution is observed off the coast of Hokkaido. (**b**) Observed sea surface temperature (unit: °C). The light grey shading north of Hokkaido represents sea ice cover areas on the day the training vessel reached the ice margin. (**c**) Sea surface temperature minus 500-hPa atmospheric temperature. The weakness of vertical stratification is indicated by this value, and a 43 °C difference is the criterion for the occurrence of a polar low^19^. Temperature differences (unit: °C) larger than 43 °C are indicated in yellowish or reddish colours. (**d**) Cloud top altitudes (unit: m).

Our air-sea observation area extended from the southern edge of the Japan Sea through the Soya-strait and entered the Okhotsk Sea (Fig. 4). During the observational period from January 19 to 27, large-scale atmospheric conditions were such that cold air advection was occurring due to a Siberian monsoon wind. Thus, the observations taken across the Japan Sea produced measurements covering a variety of air–sea interactions in the Japan Sea under cold air advection. By comparing these measurements to the occurrence of the JPCZ, specific air-sea features of the JPCZ can be revealed. Figure 4a shows a map of horizontal wind direction and speed at an altitude of 10 m on all the radiosonde launches, including in the JPCZ area in the left bottom side of the figure. Over the area off the coast of Hokkaido, a cyclonic vortex-like structure is observed. This is probably a polar low, which occasionally occurs in this region. SSTs along the observation line show several notable ocean features (Fig. 4b). One is that the JPCZ area is particularly warm in comparison to other areas. Another is that fine ocean structures smaller than 100 km are observed along the 40° N latitude. This latitudinal area is known to be a subpolar frontal zone between the warm SSTs of Tsushima Warm Current and northern cold SSTs. Another is that even off the coast of Hokkaido, SST was relatively high at about 8 °C, owing to the northward intrusion of the Tsushima Warm Current. In contrast, the SST near sea ice was almost at the freezing point of sea water and the lowest during the cruise.

Figure 4c shows the difference of SST from overlying 500-hPa atmospheric temperature, i.e., SST minus the temperature at an altitude with a pressure of 500 hPa. The degree of difference is known to be an indicator of a polar low occurrence for which a temperature difference of 43 °C is a criterion^19^. In the eastern parts of the JPCZ, off the coast of Hokkaido, and near the Tsugaru Strait, this criterion was exceeded with values of 43.5, 43.2, and 46.6 °C, respectively. In the latter two areas, vortex-like phenomena—probably polar lows—were observed. The JPCZ also exceeded the criterion for the polar low occurrence. In fact, some JPCZs are accompanied by meso-scale vortex development^24,25^. The upper air temperature at 500 hPa was on average − 28 °C in JPCZ, and − 35 and − 36 °C in the two polar vortex periods. Whether this large temperature difference was due to the presence of anomalously cold air or to the warmth of the oceans is considered next. These upper air temperatures were as low as normal climatological temperatures in these individual regions for this time of the season, so this criterion can be satisfied without having unusually cold upper air. In other words, this criterion can be satisfied under ordinary atmospheric fields. Thus, the Tsushima Warm Current plays the main role in exceeding the criterion. Therefore, the JPCZ or polar low is likely to occur in these areas with climatological atmospheric oceanic environments. The values of the SST minus the 500-hPa temperature during the ‘normal’ band-shaped cloud pattern or near the sea ice were much lower than the criterion.

Figure 4d shows the comparison of the JPCZ cloud top altitude with those of the ‘normal’ band-shaped cloud pattern and the cyclonic vortex-like pattern. The cloud top altitude was determined from the agreement between the temperature significant level and altitude of the rapid decrease in relative humidity to below 85%^20,21^. The cloud-top height was higher for the JPCZ than for others. The cloud was also high during the period with the cyclonic vortex-like structure at about 2.5 km. The highest cloud top altitude is shown just east of 140° E, 42° N where a polar low formed. Cloud tops were from about 1 to 2 km, and the average is 1.9 km between 36° N and 41° N and near the sea ice region in the ‘normal’ band-shaped cloud pattern.

**Figure 5:**
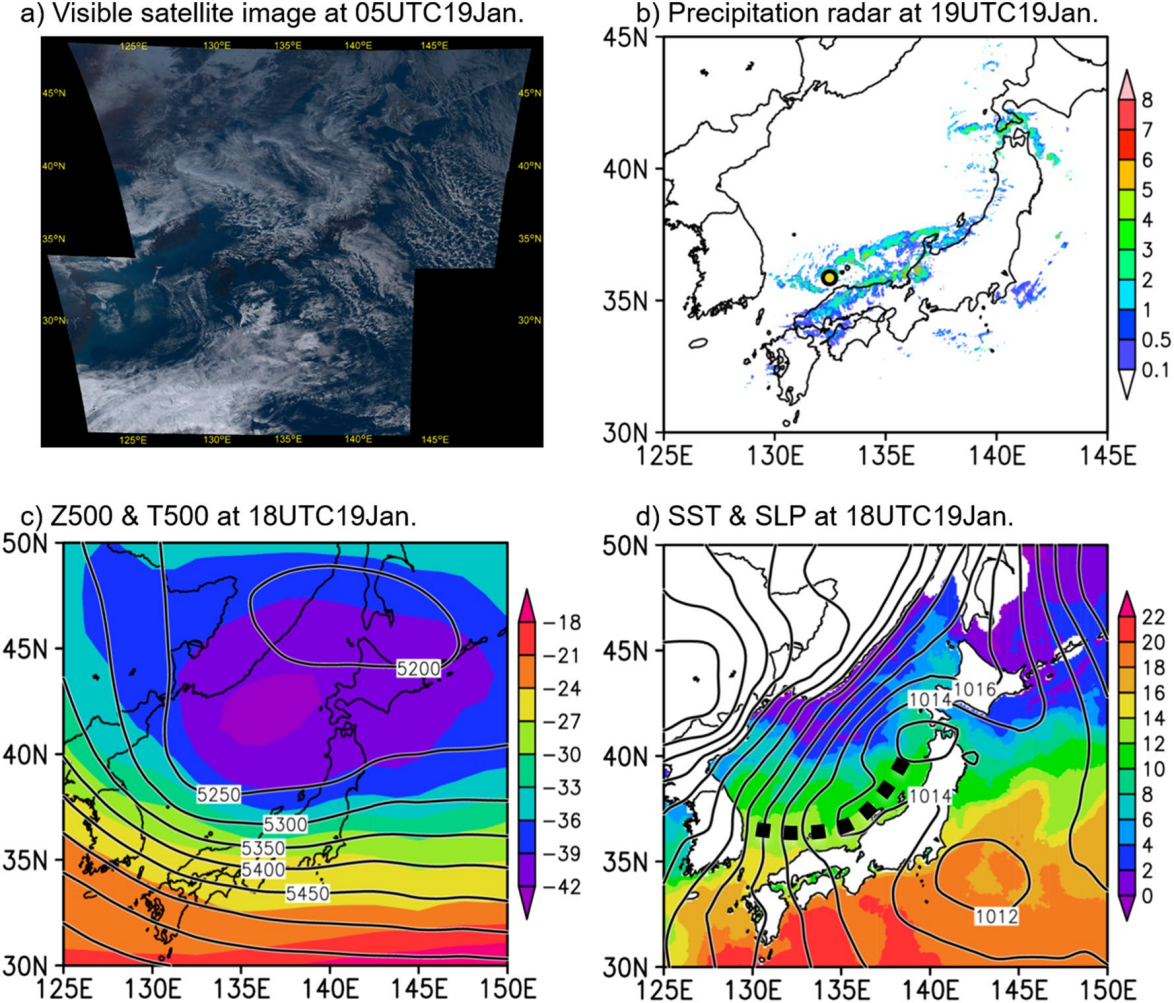
Visible satellite image, precipitation radar, surface and upper air atmospheric fields, and sea surface temperature field in the period of the JPCZ observation. (**a**) A Himawari satellite visible image at 05UTC 19 January, which corresponds to 14:00 local time. (**b**) Composite map of precipitation radar at 19 UTC 19 January, when the training ship crossed the centre of the JPCZ. A heavy circle indicates the ship location at 19 UTC 19 January. (**c**) Contours show 500 hPa geopotential height. Shadings show temperature at the level of 500 hPa at 18UTC 19 January. (**d**) Contours show sea level pressure at 18UTC 19 January, and the shadings shows sea surface temperature by HIM SST on 19 January. A dashed line indicates that a trough from a cyclone over the Tsugaru Strait elongated southwestward along the Japan Sea coast.

A Himawari satellite visible image on 05 UTC 19 January 2022 (Fig. 5a) shows a bold band-shaped cloud line, which is the JPCZ, over the Japan Sea. Figure 5b shows a combined radar image on 19UTC 19 January. The band-shaped precipitation zone, i.e., JPCZ, is recognizable on radar with a precipitation zone positioned west–east; the circle indicates the vessel position. The location of the JPCZ on radar was shifted southward compared to that on the satellite image, indicating that the JPCZ was moving southward during the observation period. The training vessel thus crossed the southward moving JPCZ, all the while we conducted radiosonde and eXpendable Conductivity Temperature depth (XCTD) measurements at 1 h intervals. The JPCZ that was initiated downstream of Mt. Paektu appeared a few days before 19 January 2022 (satellite images not shown). The JPCZ peaked on 18 January. After 19 January, the JPCZ disappeared, so the observed JPCZ was not in the peak mature phase but in a condition of decline. If the observations had captured the peak period, the results may have been more intense than those presented in this paper.

Figure 5c,d show atmosphere and SST fields on 19 January. A 500-hPa trough accompanying the upper cold air mass was located in the northern part of the Japan Sea. The sea level pressure field shows a Siberian high on the continent, whereas two meso-scale cyclones were located over northern Japan and to the east of Japan. A trough from the cyclone over the Tsugaru Strait elongated southwestward along the Japan Sea coast in central Japan in the sea level pressure field. This probably corresponds to the observed JPCZ. The SST field shows that warm area in the Japan Sea extended northward along the coast of Japan, whereas cold SSTs extended southward along the continental coast.

**Figure 6:**
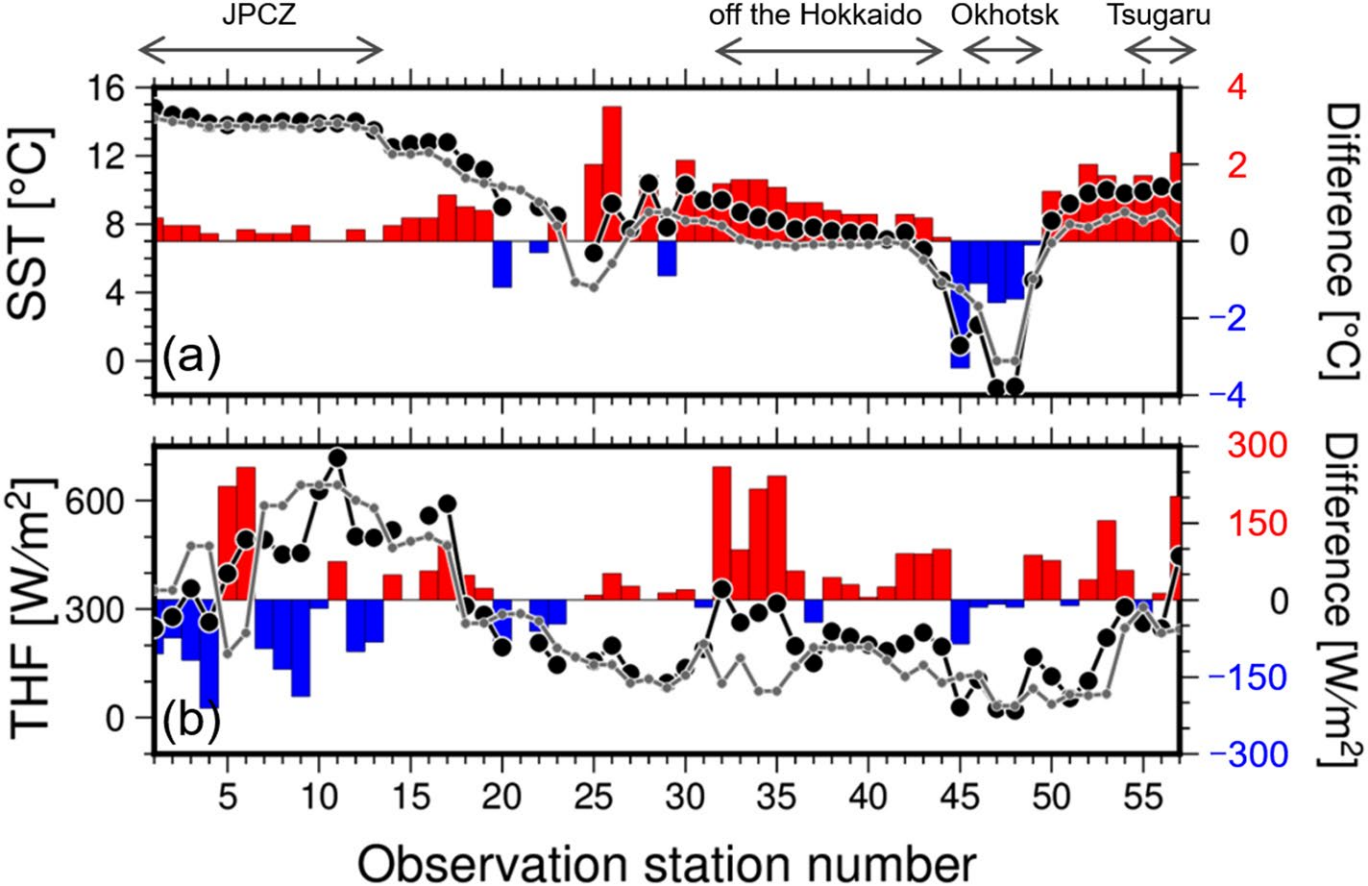
Observed SST and vertical heat flux were, on average, higher than in the atmospheric oceanic dataset by JMA. (**a**) Black filled circles and line show observed temperature and grey filled circles and line show a dataset of HIM SST data, which is used for numerical prediction by JMA. The comparison is at the nearest grid point and the nearest time to each observation station and data collection time. Positive differences mean that the observed temperature is higher than HIM SST. (**b**) Same as in (**a**) but for the sum of the sensible and latent heat flux. Heat flux by a reanalysis product data is from the JRA55 dataset^26^.

Comparison of our observational SST with a SST product, High resolution Merged satellite and in situ data Sea Surface Temperature (HIM SST), which is used as a surface boundary condition for the numerical prediction of the Japan Meteorological Agency (JMA), is shown in Fig. 6. Although there was overall good agreement, the observed SST was on average higher than for the SST product. In some areas, particularly in northern areas, the observed SST was more than 2 °C higher than the SST product. Observed SSTs near the sea ice from P45 to P48 was about 2 °C lower than the SST product. In association with this difference in SST, the surface heat flux was also different. Because the SST difference of this magnitude produces a heat flux difference of about 100 W m^−2^, numerical simulation driven by the HIM SST may underestimate the actual atmospheric phenomenon that occurs during the observation period. In fact, the difference in flux exceeded 200 W m^−2^ in the area off the coast of Hokkaido, in which a polar low formed. Differences in flux in the JPCZ area were also large, e.g., the difference exceeded 200 W m^−2^ and, moreover, the range of variability between positive and negative deviations was large. Wind speed difference between the observation and reanalysis product (not shown) was responsible for this difference because the SST difference was small in the JPCZ area. These differences remind us of the importance of direct observations.

### Comparison of observed JPCZ and numerical simulation

**Figure 7:**
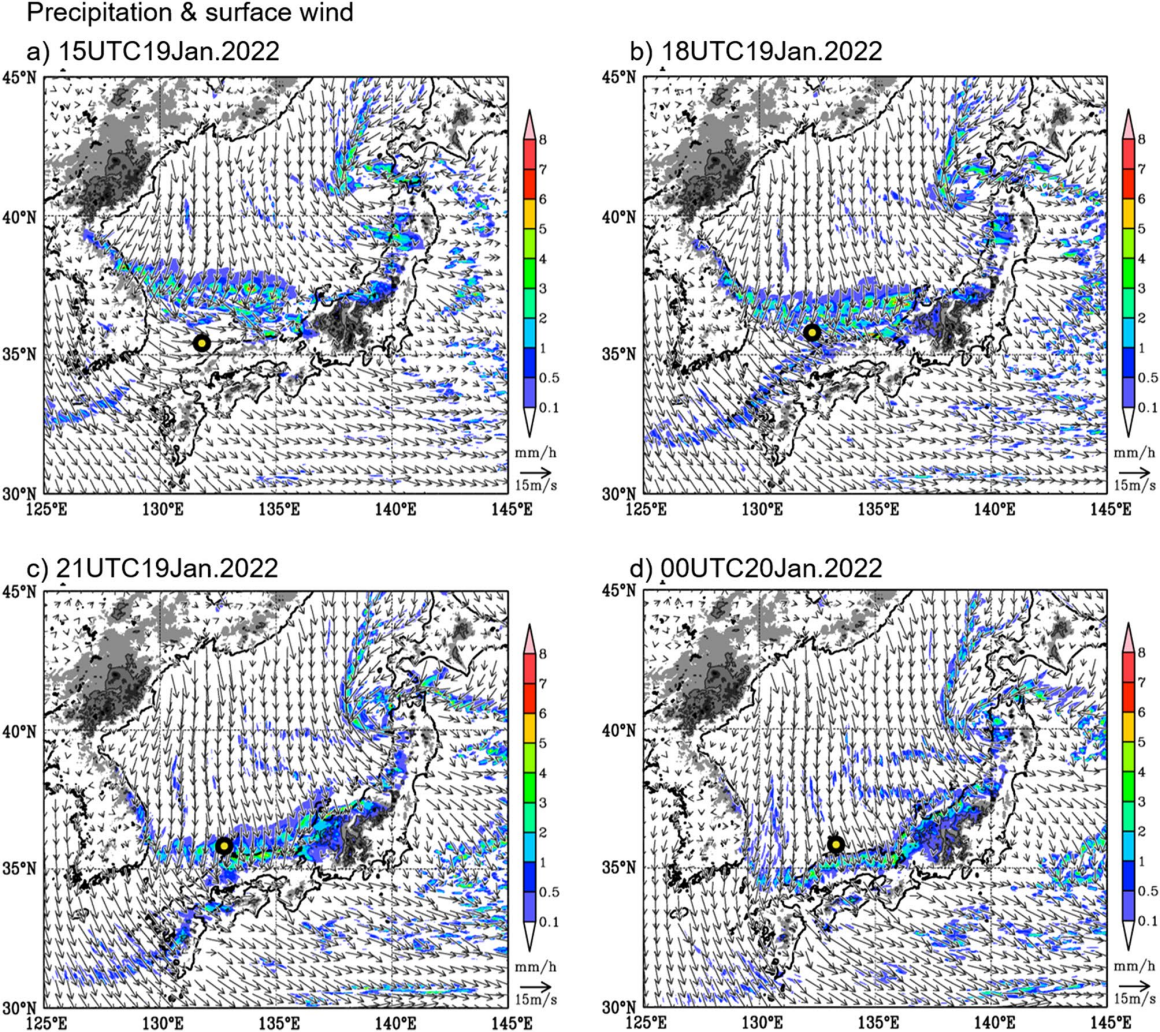
JPCZ by a numerical simulation during the observation period. Surface wind and precipitation simulated by the nonhydrostatic numerical model (NHM) of JMA at the same time as the onboard observation. Initial date is 00UTC 18 January 2022. (**a**) 15UTC 19 January. A heavy circle indicates the ship location at the time of the model results. (**b**) 18UTC 19 January, (**c**) 21UTC 19 January, and (**d**) 00UTC 20 January. Precipitation is indicated in mm/h. Grey shadings represent topography over 500 m and get dark every 500 m. The surface wind is drawn over 500 m. Contours show the elevation of 1000 m.

**Figure 8:**
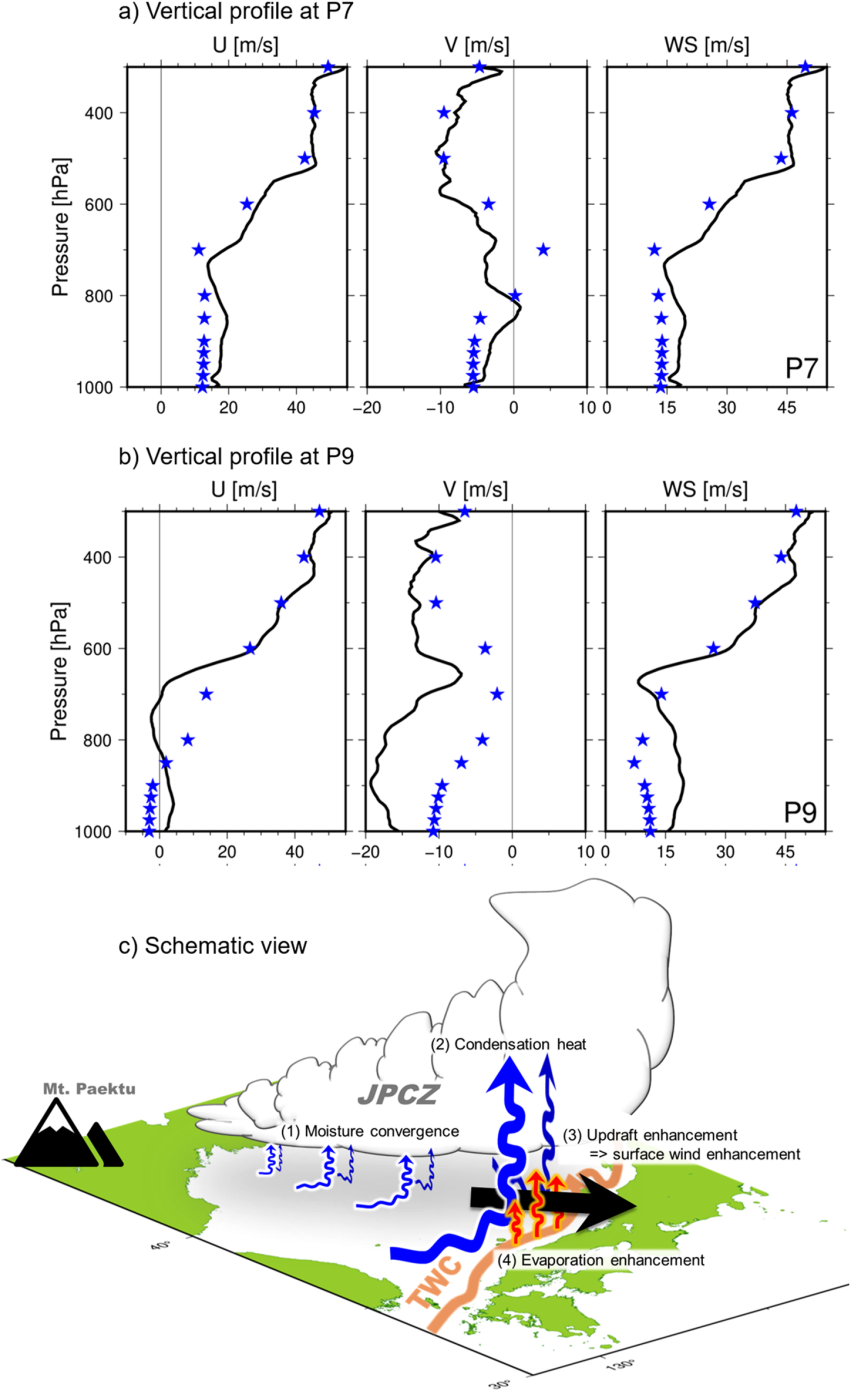
Plots of the observed winds in comparison to simulated winds near and off the centre of the JPCZ, and Schematics showing the self-sustaining mechanism of the JPCZ. (**a**) Observed west (U) and south (V) winds components at observation station P7 is shown by black line in the left and centre panels, respectively. Right panel shows wind speed. Stars indicate simulated wind at the same observation station and at the same time. P7 is located to the west of the centre of the JPCZ. (**b**) Same as in (**a**) but for observation station P9, which is near the centre of the JPCZ. (**c**) Schematic views. (1) Moisture from many branches converges to form a large moisture flow. This has a structure of an atmospheric river. (2) The JPCZ gains buoyancy from the condensation heat of the water vapour, and the convergence thus strengthens the updrafts. (3) The enhanced updrafts further strengthen the horizontal convergence of winds, i.e., enhanced wind speeds. (4) The enhanced wind speed further increases evaporation from the ocean and moisture convergence. Therefore, the JPCZ with the structure of atmospheric rivers has a self-sustaining mechanism.

Figure 7 shows the maps of horizontal surface wind and precipitation by numerical simulation from 15UTC 19 Jan to 00UTC 20 Jan. The JPCZ is well simulated. A strong precipitation zone, which corresponds with the JPCZ, extends from the root of the Korean Peninsula to Japan through its southward movement. The surface wind direction changed drastically over the JPCZ as in the observation. Based on the vessel locations denoted by the thick circles, we consider that the vessel crossed the southward moving JPCZ during this cruise. Figure 8 shows the comparison of simulated wind profiles with those of the observations at P7 and P9. P7 is located to the west of the JPCZ while P9 is near the centre. The observation profiles at P7 are overall similar to the simulation results. In contrast, at P9, simulated wind speeds are weaker than those of the observation overall, and they are markedly weaker near the surface, where simulated wind speed is less than half of the observed values. Wind direction difference near the surface is also large at P9. Although further comparisons are needed, this is outside the scope of this paper and will be a subject of future consideration. It is interesting to execute a numerical experiment by switching the SSTs to the observational ones and compare results, and this will also be a subject for future research.

### Estimation of potential snowfall from the JPCZ

Here, we roughly estimate the amount of water vapour supplied to the JPCZ from the underlying ocean and from the surrounding atmosphere. The oceanic in situ supply, i.e., the ocean surface evaporation associated with the latent heat flux, was about 0.83 kg m^−2^ h^−1^ as estimated by the bulk method^21^. On the other hand, the atmospheric supply by the horizontal moisture flux due to airflow convergence was about 13 kg m^−2^ h^−1^. This estimation is based on the observed wind convergence along the observation line and observed humidity. The total moisture supply is thus 14 kg m^−2^ h^−1^. This value is equivalent to a snow depth of 14 cm h^−1^, assuming that the density of the new snow is 0.1 g cm^−1^. Therefore, the JPCZ captured in this observation had the potential to produce snow accumulation of about 100 cm if the snowfall continues for 7 h. This value is equivalent to about 8200 W m^−2^ of condensation heat of water vapour if all the water vapour condensed in clouds. In addition, the convergence of airflow is evaluated by the same method as the moisture. The estimated convergence is 7.9 × 10^–4^ s^−1^ at the height of 100 m. Because the estimation ignores a term perpendicular to the observation line, this may be an overestimate. Wind speed in the centre of the JPCZ was weaker than in surrounding areas as shown in Fig. 2b. Without convergence, the horizontal wind speed crossing the observation line may have been strong. Thus, the degree of overestimation is not so large. Horizontal convergence by JRA55 reanalysis is about 2.0 × 10^–5^ s^−1^ at the height of 1000 hPa at the point nearest to the observation. This value is about 1/40 of the observed one.

Because water vapour formation within the JPCZ is supplied from the surrounding moist air accumulated from the ocean under the surrounding atmosphere as well as from the underlying ocean, strong surface winds around the convergence zone observed at points P6 and P11a in Fig. 2b promote evaporation and increase the water vapour supply. Since these strong winds are created by the convergence and updraft caused by the JPCZ, the JPCZ has a self-sustaining mechanism that further enhances the water vapour supply from the surrounding ocean (Fig. 8c). Some similarities between the JPCZ and atmospheric rivers are discussed in order to briefly explore this mechanism. The main source of moisture is the oceans surrounding the JPCZ, and the JPCZ plays the role of collecting moisture evaporated from those surrounding oceans. Similar horizontal convergence possibly occurs in many areas along the JPCZ from its upstream area through its downstream area because the JPCZ is considered to has a banded, elongated and two-dimensional structure^5^. This structure, in which moisture from many branches converges to form a large moisture flow, is exactly the same structure as a river. The JPCZ gains buoyancy from the condensation heat of the water vapour, and the convergence thus strengthens the updrafts. The enhanced updrafts further strengthen the horizontal convergence of winds, i.e., enhanced wind speeds. The enhanced wind speed further increases evaporation from the ocean and moisture convergence. Therefore, the JPCZ with the structure of atmospheric rivers has a self-sustaining mechanism. This mechanism suggests that once a JPCZ is formed, it may not disappear in a short period of time but rather may persist for a long time. The JPCZ is miniature in terms of atmospheric rivers because its size and amount of water vapour are smaller than those of conventional ones. Since its size is much smaller than that of an atmospheric river, it might be more appropriate to refer to it as an atmospheric creek. While the well-known atmospheric rivers transport moisture from southern warm oceanic areas to northern cold areas^7^, the JPCZ transports moisture southward.

## Conclusions

As the first simultaneous air-sea shipboard observation of the JPCZ, all measurements presented here are new. Some of the most important points are summarised below. Our observations showed that the wind direction changed drastically by about 90 degrees from the surface to about 3.5 km aloft within an extremely narrow horizontal distance of about 15 km, indicating strong airflow convergence from surrounding areas toward this narrow area. The confluence and convergence of airflows at the JPCZ, which have been identified in previous numerical simulation studies, were indeed present. The airflow convergence was quantified at about 7.9 × 10^–4^ s^−1^. Cloud top was at a height of about 3.5 km, which is nearly twice as high as ‘normal’ clouds. Surface air temperature was much lower than that of the underlying sea with strong wind speed. Large amounts of heat were thus gained by the warm sea (maximum: 718 W m^−2^). Water vapour was also concentrated on the JPCZ with horizontal air convergence with a value of about 13 kg m^−2^ h^−1^, which is equivalent to 8200 W m^−2^ condensation heat in the JPCZ. This water vapour concentration has the potential to produce snowfall with a depth of about 100 cm over 7 h. Owing to the horizontal moisture flux from the surroundings areas, the main contributions to the JPCZ cover a wide range of the surrounding area. The JPCZ can be, therefore, a kind of an atmospheric river that occurs under the influence of cold air advection over the sea. Since condensation heating in the JPCZ accelerates updrafts and horizontal convergent winds, surface evaporation from the surrounding ocean is strengthened. Therefore, the JPCZ has a self-sustaining mechanism that further enhances the water vapour supply from the oceans (Fig. 8c). In conclusion, the simultaneous quantitative observations of the atmosphere and ocean showed that warm oceans, e.g., the Tsushima Warm Current, play an important role in concentrating large amounts of water vapour in the JPCZ.

In addition to the JPCZ, polar lows were observed in the northern Japan Sea. A common feature of the polar lows and the JPCZ was that the difference between SST and 500-hPa temperature was quite large, exceeding 43 °C, and that the value was as large as the climatological normal temperature difference. Northward intrusion of the Tsushima Warm Current contributes to this difference. We can therefore propose that the Tsushima Warm Current is responsible for the frequent occurrence of such extreme clouds as the JPCZ and polar lows that form in the Japan Sea. Since these observations alone are not sufficient for discussing climate, we aim to present our observations and analysis of the air-sea interaction to make it available for further observations and discussion and to contribute to improving the forecasting of JPCZ events.

## Methods

### Observations

We conducted simultaneous air-sea observations in the Japan Sea from the training vessel, Koyo-maru of National Fisheries University, from January 19 to 27, 2022. The cruise route shown in Fig. 1 was from the southern edge of the Japan Sea to the northern edge of Hokkaido and included entry of the sea-ice area in the Okhotsk Sea. Meteorological balloons, radiosondes, were launched during the observations. The radiosondes measured atmospheric vertical profiles of pressure, temperature, relative humidity, and wind from the surface to an altitude of the tropopause. We launched radiosondes at intervals of 1 h in the JPCZ and areas where vortexes occur. In other locations, we conducted launches every 3 or 6 h. As it took the radiosonde slightly longer than 1 h to reach the tropopause, three sets of radiosonde receiving instruments were utilised on the training vessel for these observations. XCTD measurements were also carried out at the same time as the radiosonde launches to measure vertical profiles of temperature and salinity in the ocean.

Due to strong winds near the centre of the JPCZ, such as at P9, several balloons needed to be relaunched and were referenced as P9a, P9b etc. As data from temperature and humidity sensors were missing on these launches, but GPS data and associated wind data were collected, fine time-resolution data on vertical profiles of wind could be obtained at all points.

### Model estimation of water vapour contribution

Estimation of the amount of water vapour supplied to the JPCZ from two sources was considered. One is from the ocean under the JPCZ, and the other is from the surrounding atmosphere. The water vapour supplied from the sea under the JPCZ was estimated by the amount of evaporation from the sea surface using the bulk method of CORE3.0a^22^ based on observed surface wind speed, SST surface air temperature, and relative humidity. Horizontal moisture flux convergence, i.e., the water vapour supplied from the surroundings, was estimated by

1 $$-{\int }_{0}^{h}\frac{\delta \left(\rho qV\right)}{\delta d}dz,$$

where $\delta d$ is the distance between two adjacent observation points, $\rho$ is air density, and $q$ is observed water vapour mixing ratio. $V$ is a wind component parallel to a line connecting the adjacent two points. $\delta (\rho qV)$ is the difference in moisture flux along the line. $h$ is the height to which a drastic wind direction change was observed and was limited to 3500 m.

### Data

The Japanese 55-year Reanalysis (JRA-55) data^26^ and high-resolution merged satellite data and in situ Sea Surface Temperature (HIM SST) data (https://ds.data.jma.go.jp/gmd/goos/data/rrtdb/jma-pro/him_sst_pac_D.html) were used to produce atmospheric and oceanic fields, and HIM SST was also used to make calculations for comparing to the SST observations. A Himawari satellite visible image obtained for 05 UTC 19 January 2022 and a combined radar image obtained on 19UTC 19 January were both provided from JMA.

### Numerical simulation

Numerical simulation was conducted using the JMA Nonhydrostatic Model^27^ (NHM). The mesoscale gridded analysis data derived from the JMA and HIM SST were used for the initial and lateral boundary conditions of the NHM. The horizontal resolution was 2 km grid spacings. The NHM is a z* hybrid coordinate with 50 vertical layers. The model bottom and top heights were 40 and 20,000 m, respectively, the model domain was the whole Japan Sea, and the initial date was 00Z 18 January 2022. The physical processes of precipitation and atmospheric boundary layer were calculated by the bulk-type cloud microphysics^28^ and by the improved Mellor-Yamada-Nakanishi-Niino (MYNN) Level 3 model^29^, respectively. An ordinary simple slab scheme was used for a land surface process. The surface bulk coefficients were from Beljaars and Holtslag^30^.

## Data Availability

All observation data are available at ‘https://atm.bio.mie-u.ac.jp/JPCZ/data.html’. The analysed datasets along with the initial and boundary conditions of the numerical experiments are open to the public as indicated in the Methods section. All figures were generated using following open software: GMT (Version 6.2.0; https://docs.generic-mapping-tools.org/dev/index.html), ODV (Version 5.6.2; Schlitzer, Reiner, Ocean Data View, https://odv.awi.de, 2022), and GrADS (Version 2.2.1; http://cola.gmu.edu/grads/grads.php).
